# Optimization of number and range of shunt valve performance levels in infant hydrocephalus: a machine learning analysis

**DOI:** 10.3389/fbioe.2024.1352490

**Published:** 2024-03-18

**Authors:** Mark Graham Waterstraat, Arshia Dehghan, Seifollah Gholampour

**Affiliations:** Department of Neurological Surgery, University of Chicago, Chicago, IL, United States

**Keywords:** hydrocephalus, shunt surgery, valve performance level, valve drainage pressure, cerebrospinal fluid, intracranial pressure, infant, machine learning

## Abstract

Shunt surgery is the main treatment modality for hydrocephalus, the leading cause of brain surgery in children. The efficacy of shunt surgery, particularly in infant hydrocephalus, continues to present serious challenges in achieving improved outcomes. The crucial role of correct adjustments of valve performance levels in shunt outcomes has been underscored. However, there are discrepancies in the performance levels of valves from different companies. This study aims to address this concern by optimizing both the number and range of valve performance levels for infant hydrocephalus, aiming for improved shunt surgery outcomes. We conducted a single-center cohort study encompassing infant hydrocephalus cases that underwent initial shunt surgery without subsequent failure or unimproved outcomes. An unsupervised hierarchical machine learning method was utilized for clustering and reporting the valve drainage pressure values for all patients within each identified cluster. The optimal number of clusters corresponds to the number of valve performance levels, with the valve drainage pressure ranges within each cluster indicating the pressure range for each performance level. Comparisons based on the Silhouette coefficient between 3-7 clusters revealed that this coefficient for the 4-cluster (4-performance level) was at least 28.3% higher than that of other cluster formations in terms of intra-cluster similarity. The Davies-Bouldin index for the 4-performance level was at least 37.2% lower than that of other configurations in terms of inter-cluster dissimilarity. Cluster stability, indicated by a Jaccard index of 71% for the 4-performance level valve, validated the robustness, reliability, and repeatability of our findings. Our suggested optimized drainage pressure ranges for each performance level (1.5–5.0, 5.0–9.0, 9.0–15.0, and 15.0–18.0 cm H_2_O) may potentially assist neurosurgeons in improving clinical outcomes for patients with shunted infantile hydrocephalus.

## Introduction

Hydrocephalus is a complex central nervous system (CNS) disorder characterized by an abnormal accumulation of cerebrospinal fluid (CSF). Although not a disease in itself, hydrocephalus manifests as a condition with diverse underlying causes. Congenital hydrocephalus exhibits a prevalence with rates of 50–70 per 100,000 births in North America, 80–110 in Europe, and up to 750 per 100,000 in Africa (JH, 2005; Warf, 2005; Munch et al., 2012; Dewan et al., 2018). The main treatment for hydrocephalus involves shunt surgery, a procedure designed to divert excess CSF and alleviate intracranial pressure (ICP) (Gholampour et al., 2019). Hydrocephalus is the leading reason for brain surgery in children, making it the most common condition requiring surgical intervention (Ponomaryov and Merkulova, 2015; Duy et al., 2023). Long-term evaluations have indicated that more than 85% of pediatric hydrocephalus patients may suffer shunt failure (Reddy et al., 2012). Infant patients show an even greater failure risk and lower shunt survival rates at all periods compared to older pediatric peers (McGirt et al., 2002; Shannon et al., 2012; Riva-Cambrin et al., 2016). Therefore, optimizing the functionality of the shunt system can be crucial to enhancing shunt efficiency and reducing shunt failure, particularly in the case of infant hydrocephalus.

Previous studies have attempted to consider the impact of various variables to reduce shunt failure and improve shunt outcomes. In this context, we evaluated the effect of different catheter routes on the risk of shunt failure (Gholampour et al., 2022b). Additionally, several studies have examined the impact of valve characteristics on shunt efficacy. Khan et al. and Gruber et al. provided compelling evidence on the substantial role of antisiphon devices of valves in reducing the complications of overdrainage following shunt surgery in infant and pediatric hydrocephalus patients (Gruber et al., 1984; Khan et al., 2010). Many studies have also compared the efficacy of various programmable and non-programmable shunt valves in pediatric and neonatal hydrocephalus, demonstrating a higher efficacy of programmable valves, specifically proGAV (McGirt et al., 2005; McGirt et al., 2007; Thomale et al., 2013; Beez et al., 2014; Hall et al., 2021b). It is worth mentioning that previous studies have shown that among these shunt valve characteristics, the valve drainage pressure setting, particularly the valve performance level, plays a crucial role in reducing the shunt failure rate and improving shunt outcomes in infant hydrocephalus (Hall et al., 2021a; Bock et al., 2021). The performance level of a valve in a hydrocephalus shunt pertains to the regulatory capacity of the shunt valve, allowing neurosurgeons to adjust the pressure or flow performance level. Adjusting the valve performance level presents a significant opportunity to control the amount of CSF to be withdrawn from the ventricular system and subarachnoid space. Furthermore, this adjustment can be effective in regulating the velocity of CSF drainage, thereby reducing shunt failure specifically by preventing overdrainage or underdrainage (Gölz et al., 2013). It can also control CSF drainage pressure, which, in turn, indirectly regulates ICP, the most important variable in the management of children’s hydrocephalus (Haridas and Tomita, 2017).

Table 1 presents comprehensive specifications of shunt valves, including manufacturers, models, types, pressure ranges, numbers and ranges of performance levels, and error margins of the most renowned manufacturers of shunt valves. Notably, there is significant variability in the adjustability of shunt valves, with differences observed in both the number (from 2 to 21) and range (from 1 to 6 cm H_2_O) of performance levels across various valve models (Table 1). Yamada et al. indicated that a change in valve drainage pressure of 2–4 cm H_2_O could optimize symptom improvement (Yamada et al., 2022). Conversely, Zemack et al. contended that a minimal valve adjustment of less than 2 cm H_2_O could improve clinical outcomes; thus, considering a performance level range below 2 cm H_2_O is rational to improve shunt outcomes (Zemack et al., 2003). To address these conflicts, efficient thresholds should be found to optimize the number and range of valve performance levels, based on their closer correlation with the improvement of shunt outcomes.

**TABLE 1:** Specifications of shunt valve models, manufacturers, types, performance levels, and pressure ranges and errors (Francel et al., 1999; Chong et al., 2002; Lollis et al., 2010; Czosnyka et al., 2013; Mirone et al., 2019; Skalický et al., 2022). All pressure units are cm H_2_O.

Valve model	Valve manufacturer	Valve type	Number of performance levels	Full pressure range (min to max)	Min-max pressure in each level	Pressure range in each level	Maximum error ±
Strata II	Medtronic	Programmable	5	14 (1.5–15.5)	1.5–2.5; 3.5–5.5; 7.0–9.0; 10.5–12.5; 13.5–15.5	1; 2; 2; 2; 2	1.00
Strata NSC	Medtronic	Programmable Anti-siphon	5	20.5 (1.5–22.0)	1.5–3.5; 3.5–5.5; 9.0–11.0; 14.5–16.5; 20.0–22.0	2; 2; 2; 2; 2	1.00
Strata MR II	Medtronic	Programmable	5	14 (1.5–15.5)	1.5–2.5; 3.5–5.5; 7.0–9.0; 10.5–12.5; 13.5–15.5	1; 2; 2; 2; 2	1.00
Delta	Medtronic	Non-programmable	4	11 (1.5–12.5)	1.5–2.5; 3.5–5.5; 7.0–9.0; 10.5–12.5	1; 2; 2; 2	1.00
CSF Flow Control	Medtronic	Non-programmable	4	16 (1.0–17.0)	1.0–3.0; 3.0–4.5; 8.5–10.5; 14.5–17.0	2; 1.5; 2; 2.5	1.25
Certas Plus	Integra	Programmable	8	38 (2.0–40.0)	2.0–2.5; 4.4–5.5; 7.5–8.0; 11.0–12.0; 14.0–14.5; 17.0–18.0; 22.0–22.5; 40	0.5; 1.1; 0.5; 1.0; 0.5; 1.0; 0.5	0.5
Medos-Hakim^a^	Codman	Programmable	18	17 (3.0–20.0)	3.0; 4.0; 5.0; 6.0; 7.0; 8.0; 9.0; 10.0; 11.0; 12.0; 13.0; 14.0; 15.0; 16.0; 17.0; 18.0; 19.0; 20.0	N/A	N/A
Hakim Precision	Codman	Non-programmable	5	14 (0.0–14.0)	0.0–2.0; 3.0–5.0; 6.0–8.0; 9.0–11.0; 12.0–14.0	2; 2; 2; 2; 2	1.00
Uni-Shunt	Codman	Non-programmable	3	12 (2.0–14.0)	2.0–5.0; 5.0–9.0; 9.0–14.0	3; 4; 5	2.50
Integra DP	Integra	Non-programmable	5	21.5 (1.5–23.0)	1.5–4.0; 4.0–8.0; 8.0–12.0; 12.0–17.0; 17.0–23.0	2.5; 4; 4; 5; 6	3.00
Atlas	Integra	Non-programmable	5	21.5 (1.5–23.0)	1.5–4.0; 4.0–8.0; 8.0–12.0; 12.0–17.0; 17.0–23.0	2.5; 4; 4; 5; 6	3.00
Contour-Flex	Integra	Non-programmable	3	17 (1.0–18.0)	1.0–7.0; 6.5–12.5; 12.0–18.0	6; 6; 6	3.00
Novus	Natus	Non-programmable	2	10.5 (0.5–11.0)	0.5–5.0; 5.1–11.0	4.5; 5.9	2.95
Pedi-GAV^a^	Aesculap	Non-programmable Anti-siphon	2	5 (4.0–9.0)	4.0; 9.0	N/A	N/A
Pro-GAV^a^	Aesculap	Programmable Anti-siphon	21	20 (0.0–20.0)	0.0; 1.0; 2.0; 3.0; 4.0; 5.0; 6.0; 7.0; 8.0; 9.0; 10.0; 11.0; 12.0; 13.0; 14.0; 15.0; 16.0; 17.0; 18.0; 19.0; 20.0	N/A	N/A
m.Blue^a^	Aesculap	Non-programmable Anti-siphon	4	15 (0.0–15.0)	0.0; 5.0; 10.0 15.0	N/A	N/A
Polaris SPV^a^	Sophysa	Programmable	5	17 (3.0–20.0)	3.0; 7.0; 11.0; 15.0; 20.0	N/A	N/A
Polaris SPV-140^a^	Sophysa	Programmable	5	13 (1.0–14.0)	1.0; 4.0; 8.0; 11.0; 14.0	N/A	N/A
Polaris SPV-300^a^	Sophysa	Programmable	5	25 (5.0–30.0)	50.0; 10.0; 15.0; 22.0; 30.0	N/A	N/A
Polaris SPV-400^a^	Sophysa	Programmable	5	32 (8.0–40.0)	8.0; 15.0; 23.0; 33.0; 40.0	N/A	N/A
Equiflow	Radionics	Non-programmable	4	13 (4.0–17.0)	4.0–8.5; 7.0–11.5; 9.5–14.5; 13.0–17.0	4.5; 4.5; 5; 4	2.50

^a^ indicates valve models with no reported pressure ranges in their manufacturer’s catalog.

Medical machine learning has surfaced as a sophisticated approach to addressing classification, prediction, and optimization challenges, and it could prove useful in addressing our aforementioned concern. The comprehensive evaluation of valve characteristics requires concurrent consideration of the effects of all variables, such as anti-siphon mechanisms, programmable/non-programmable conditions, number of valve adjustments, and valve performance levels. Moreover, the multivariate nature of hydrocephalus gives rise to intricate and interrelated associations among these variables. Machine learning offers a unique opportunity to analyze the simultaneous impacts of all these variables, considering their complex interconnections. However, previous studies that have considered shunt valve characteristics in machine learning have been significantly scarce (Hale et al., 2021). This study seeks to bridge the research gap related to optimizing and standardizing valve performance level information. The goals of the present study are to establish the optimized number of shunt valve performance levels and to delineate the optimized valve drainage pressure ranges corresponding to each performance level through unsupervised machine learning analysis. This study is designed to utilize its findings to potentially improve the efficacy of shunt performance in treating hydrocephalus patients who were under 1 year of age at the time of their initial shunt surgery.

## Materials and methods

The study design, protocols, and procedures were approved by the Human Institutional Review Board (IRB) committee and adhered to the ethics guidelines of the University of Chicago (IRB code: IRB23-1157), based on the 1964 Helsinki Declaration and its later amendments.

### Study design

Several studies have highlighted the critical importance of achieving clinical improvement without shunt failure, especially following the first shunt surgery, for the treatment of infant hydrocephalus (Hall et al., 2021a; Bock et al., 2021). Hence, this study exclusively focused on infant hydrocephalus patients who demonstrated improvement after their first shunt surgery, with no documented shunt failures. This focus was based on our assumption that the findings of the present study could be leveraged to replicate these improved outcomes after first shunting for new patients. The foundation of this study lies in the categorization (clustering) of patients in our database based on their valve performance levels. To achieve this, we initially employed unsupervised machine learning clustering analysis to identify the optimal number of clusters (performance levels) within our database, considering patient characteristics, etiology, shunt characteristics, and surgical reports. Following the determination of the optimal number of clusters, we subsequently reported the maximum and minimum valve drainage pressure ranges associated with each identified cluster. It is crucial to emphasize that the number of clusters aligns with the distinct number of valve performance levels. Furthermore, the valve drainage pressure ranges within each cluster define the pressure range for each respective valve performance level.

It is worth mentioning that some studies have highlighted the significant importance of the initial adjustment of shunt valves post-surgery, particularly in influencing the clinical outcomes of hydrocephalus patients (Kim et al., 2009; Bock et al., 2021). On the other hand, some studies, while acknowledging the necessity of potential additional adjustments following the initial valve adjustment, have underscored the importance of the number of shunt valve adjustments in improving shunt outcomes in infant hydrocephalus (Cattani et al., 2023; Hall et al., 2023). Consequently, we have included both the initial valve performance level and the number of valve adjustments following the first shunt surgery in our list of features for the machine learning analysis.

### Patients’ selection and data collection

**FIGURE 1:**
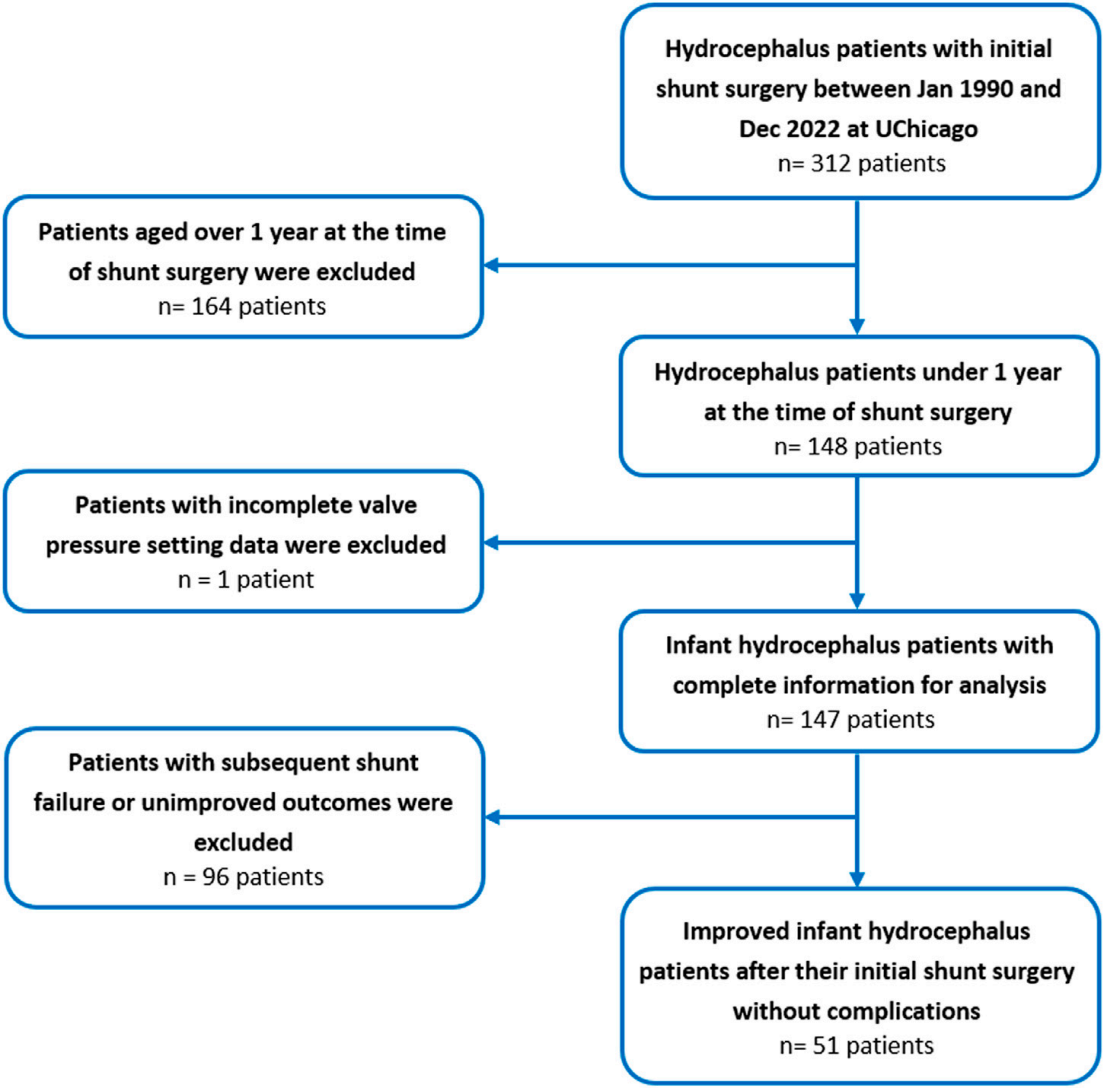
Flow chart of the study population selection including patient recruitment and exclusion criteria for infant hydrocephalus patients who underwent the first shunt surgery between January 1990 and December 2022 at the University of Chicago.

This single-center, retrospective cohort study included all infant hydrocephalus patients who underwent shunt surgery and did not experience subsequent failure or unimproved outcomes at the University of Chicago Medicine between January 1990 and December 2022 (Figure 1). The primary criteria for diagnosing and selecting cases of hydrocephalus were clinical signs and symptoms in patients, such as enlarged head size, bulging fontanelle, poor feeding, vomiting, sleepiness or lethargy, developmental delays, and physical symptoms. Imaging was also used for the diagnosis of some patients. The initial cohort was generated from the EPIC database, an electronic health record system. Inclusion criteria were (i) patients treated operatively with a shunt for hydrocephalus, (ii) absence of subsequent shunt complications, failure, unimprovement outcomes, or revisions, (iii) the first shunt surgery conducted before the patient reached 12 months of age, (iv) clear and complete recording of the clinical data, surgical variables, and initial shunt valve’s pressure setting information, (v) no history of any other neurosurgical surgeries. The study encompassed all first shunt surgeries, including ventriculoperitoneal, -pleural, and -atrial placements, while excluding shunt removals without replacement. Patients who underwent their first shunt surgery outside of the University of Chicago and subsequently came to the University of Chicago for shunt revisions were excluded from this study. It is worth mentioning that we reviewed the medical documents of all patients to ensure there were no reports of unimproved clinical outcomes after the first surgery. To define the shunt, we considered factors leading to shunt revision or removal of the initial shunt, such as mechanical failures, infections, leakage, underdrainage, overdrainage, or blockages. To determine the unimproved outcomes of the shunt surgeries, we followed up with the patients on their EPIC documents. During these follow-ups, we assessed the presence or absence of persistent or worsening signs and symptoms. In some cases, imaging was also used in conjunction with clinical signs and symptoms to make decisions about improvement. Previous studies have established the crucial role that increased ICP plays in the development of hydrocephalus and its clinical implications (Gholampour et al., 2017b; Gholampour et al., 2017a; Gholampour, 2018; Gholampour and Fatouraee, 2021). Recent research supports this view and has shown that pressure-regulated valves are more effective in reducing the risk of shunt revision and obstruction compared to flow-regulated valves in neonates with hydrocephalus (Mulcahy and Ma, 2023). Consequently, in the present study, exclusively pressure-regulated valves were utilized.

Operative reports, medical profiles, and post-operative follow-up evaluations were reviewed for analysis. Patients “age at shunting, patients” gender (male or female), hydrocephalus etiology (six etiologies), and type of hydrocephalus (communicating or non-communicating), were reported and analyzed. Additionally, surgical variables such as shunt catheter route (frontal, parietal, or proximal), anti-siphon status, programmable status, number of valve adjustments, and valve performance level were collected from operative reports. These variables were employed in unsupervised machine learning analysis to optimize the number and range of valve performance levels. It should be noted that shunt valve performance levels were also converted to drainage pressure values according to the respective manufacturer’s catalogs when specific drainage pressures were not recorded in the operative reports (Francel et al., 1999; Chong et al., 2002; Lollis et al., 2010; Czosnyka et al., 2013; Mirone et al., 2019; Skalický et al., 2022).

It should be noted that six categories of hydrocephalus etiology were defined: brain and skull malformations, spinal disorders, cysts or tumors, post-hemorrhage, post-infection, and congenital or idiopathic. Brain and skull malformations included aqueductal stenosis, schizencephaly, hydranencephaly, macrocephaly, agenesis of the corpus callosum, and partial agenesis of the frontal and temporal lobes. Spinal disorders included in the study were Chiari malformation type I and myelomeningocele. Cysts and tumors included Dandy-Walker cysts, intracranial abscesses, and subdural hematomas. Post-hemorrhagic etiologies included intraventricular and subdural hemorrhages. Post-infectious causes for hydrocephalus were meningitis and cytomegalovirus.

### Machine learning analysis

In the present study, we employed unsupervised hierarchical clustering algorithms using Python 3.11 software to categorize analogous records within our database into defined clusters. The features utilized for clustering included patient age and gender, type of hydrocephalus (two types), hydrocephalus etiology (categorized into six main groups with more than 20 sub-groups), shunt catheter route (three routes), shunt anti-siphon status (two status), programmable status (two status), number of valve adjustments, and valve drainage pressure. Hierarchical clustering was executed utilizing the Euclidean distance metric to determine the dissimilarity between data points. This approach structured the data into a hierarchical tree alignment, whereby clusters were iteratively amalgamated based on their similarity. Commencing with each datum as a distinct cluster, the algorithm successively merged the pair of clusters exhibiting the greatest similarity. This iterative merging persisted until the culmination in a singular, all-encompassing cluster. The similarity between clusters was determined by a linkage method. To facilitate this hierarchical clustering, the “linkage”, “dendrogram”, and “fcluster” functionalities within the “SciPy” library of Python were employed. It is worth mentioning that the Silhouette coefficient for each data point *(i)* was calculated using Eq. 1 from the Scikit-learn library to evaluate the clustering quality (Nidheesh et al., 2020).

$$S\left(i\right)=\frac{b\left(i\right)\;-\;a\left(i\right)}{\max\left\{a\left(i\right),b\left(i\right)\right\}}$$ (1)

where *S(i)* is Silhouette coefficient, *a(i)* is the average distance from the *i* ^th^ data point to the other data points in the same cluster, and *b (i)* is the smallest average distance from the *i* ^th^ data point to the data points in a different cluster, minimized over clusters to which *i* ^th^ does not belong. The mean Silhouette coefficient of the clustering solution, which represents the average of the Silhouette coefficients for each data point, serves as an indicator of clustering validity. A high coefficient signifies that data points are appropriately matched to their own cluster and distinctly separated from adjacent clusters. In addition to the Silhouette coefficient, the performance of the hierarchical clustering algorithm was also evaluated using the Davies-Bouldin (DB) index. This index was computed for the set of clusters (denoted by *(* $C_{i}$ *)*) utilizing Eq. 2 from the Scikit-learn library (Zeng and Garcia-Frias, 2006).

$$DB=\frac{1}{N}\sum_{i=1}^{N}\max_{j\neq i}\left(\frac{\text{similarity}\left(C_{i},C_{j}\right)+\text{similarity}\left(C_{j},C_{i}\right)}{\text{dissimilarity}\left(C_{i},C_{j}\right)}\right)$$ (2)

where *N* is the number of clusters, $\text{similarity }\left(C_{i},C_{j}\right)$ is a measure of similarity between clusters $C_{i}$ and $C_{j}$ , and d $\text{issimilarity }\left(C_{i},C_{j}\right)$ is a measure of dissimilarity between clusters $C_{i}$ and $C_{j}$ based on the distance. It is important to note that a lower DB index value is indicative of clusters being more compact and better separated, which is desirable in cluster analysis.

It is worth mentioning that for numerical features (age, valve drainage pressure, and number of valve adjustments), we computed the mean and standard deviation. The *t*-test and one-way analysis of variance from the SciPy library in Python were employed for the statistical analysis of these numerical features. A significance threshold of *p* < 0.05 was considered.

## Results

**FIGURE 2:**
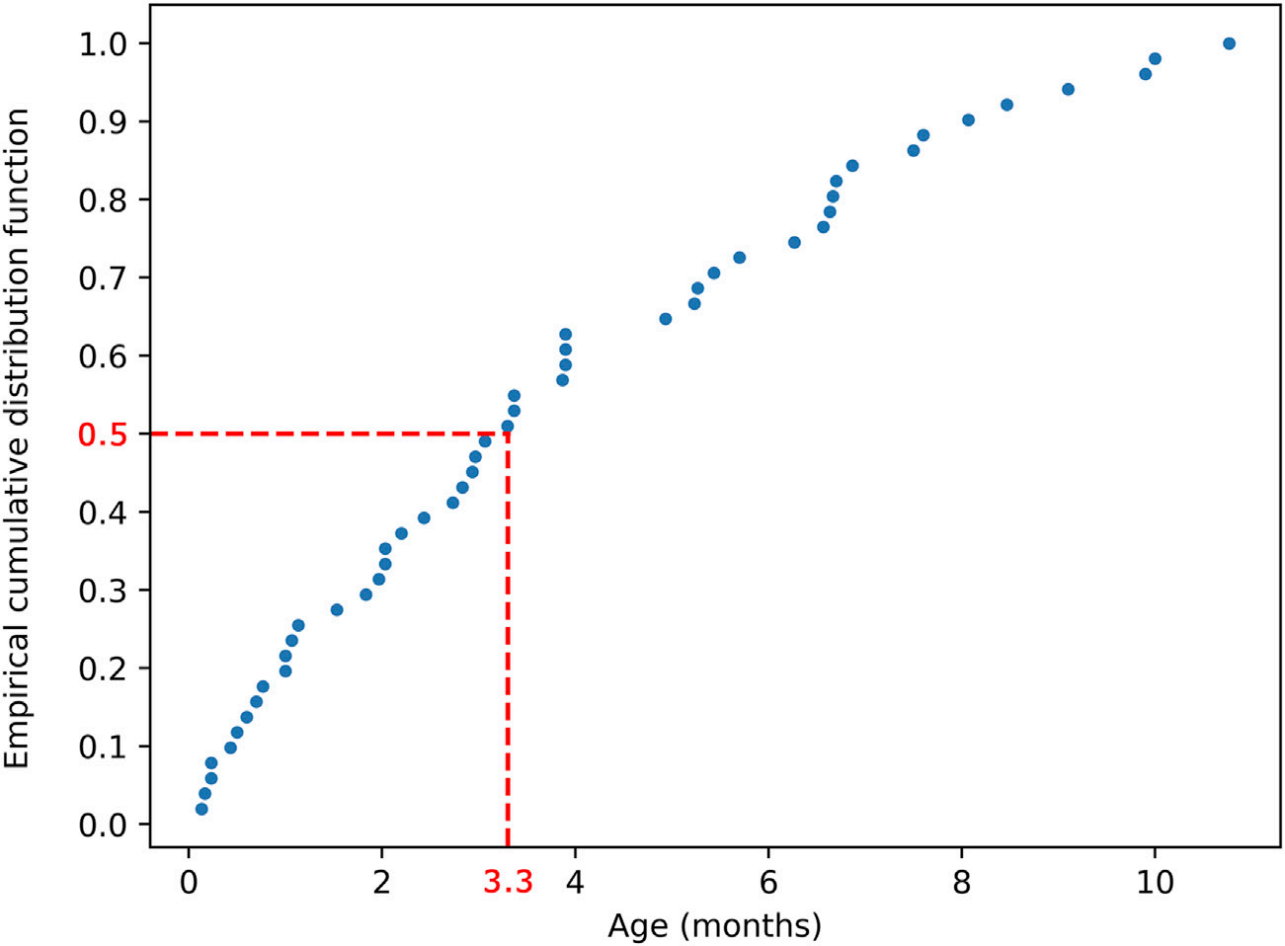
Empirical cumulative distribution function plot illustrating the age distribution of the 51 patients. The plot demonstrates that 50% of patients were aged less than 3.3 months.

The present study only focused on infant hydrocephalus patients with first shunt surgery without any failure or complications to find the best clustering based on shunt surgeries with improved outcomes. An initial cohort of 148 hydrocephalus patients aged less than 1 year with a history of shunting was identified (Figure 1). 97 patients were excluded from analysis due to subsequent shunt infection, revision, failure, unimproved outcomes, or incomplete data. This resulted in a final cohort of 51 infant hydrocephalus patients who underwent shunt surgery without subsequent failure or unimproved outcomes (Figure 1). Table 2 indicates the baseline characteristics of the infant hydrocephalus patient cohort used in the present study. The shunt failure rate for the initial shunt surgery of our patients was 65.3%. To understand the age demographics of our patient cohort, we utilized an empirical cumulative distribution function plot (Figure 2). This plot provides a visual representation of the cumulative distribution of ages among the patients. Remarkably, the analysis revealed a pivotal insight: 50% of our patients were aged less than 3.3 months. This finding underscores the significance of early-age patients within our study, shedding light on a critical demographic aspect that might influence various aspects of our analysis, from disease prevalence to treatment strategies. To verify improvements in clinical outcomes post-shunt surgery, patient documents were monitored on EPIC, with an average follow-up period of 94.9 months subsequent to the initial shunt procedure, as indicated in Table 2.

**TABLE 2:** Baseline characteristics of infant hydrocephalus patients underwent shunt surgery.

Parameters	Values	Number (%)
Age (months)	Median = 3.3, Mean = 3.9 ± 2.9	—
Gender	Male	27 (52.9%)
	Female	24 (47.1%)
Catheter route	Occipital	31 (60.8%)
	Frontal	15 (29.4%)
	Parietal	5 (9.8%)
Type of hydrocephalus	Communicating	33 (64.7%)
	Non-communicating	18 (35.3%)
Programmable valve	Yes	32 (62.7%)
	No	19 (37.3%)
Anti-siphon	Yes	15 (29.4%)
	No	36 (70.6%)
Etiology	Post-hemorrhagic	28 (54.9%)
	Brain/Skull Malformation	8 (15.7%)
	Spinal Disorder	6 (11.8%)
	Cyst/Tumor	4 (7.8%)
	Post-Infectious	3 (5.9%)
	Congenital/Idiopathic	2 (3.9%)
Valve drainage pressure (cm H_2_O)	Median = 8.0, Mean = 8.9 ± 4	—
Follow up time (months)	Median = 78.7, Mean = 94.9 ± 67.9	—

**FIGURE 3:**
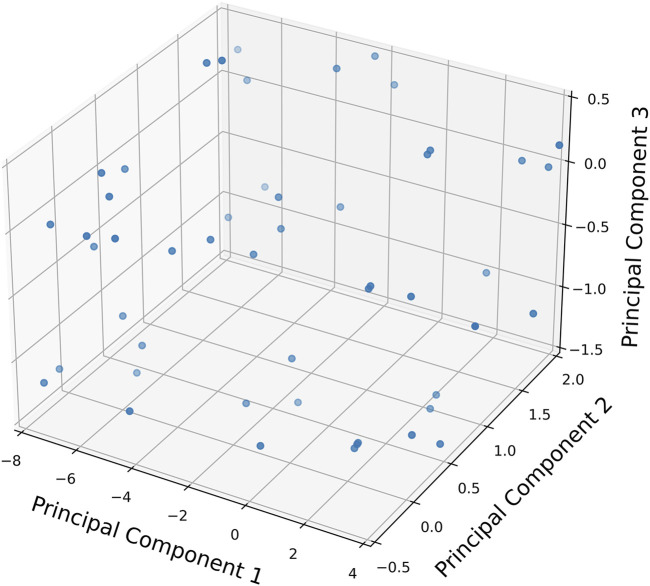
Three-dimensional principal component analysis (PCA) plot representing the dataset of 51 patients and the features. Each point denotes a patient, positioned in the 3-D space based on their combined features.

A three-dimensional principal component analysis (PCA) was performed on our dataset of patients and their corresponding features (Figure 3). This analysis reduced the dataset’s dimensionality while preserving variance, allowing for a nuanced exploration of complex patient data. Patients are depicted as points in a 3D space, with their positions determined by their combined features.

**FIGURE 4:**
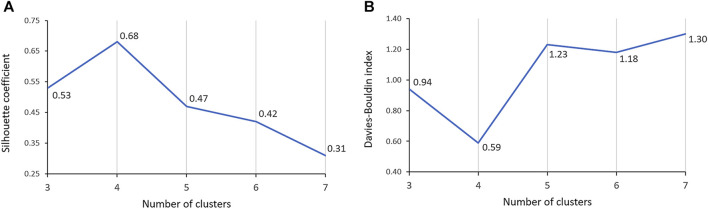
Comparison of Silhouette coefficient **(A)** and Davies-Bouldin index **(B)** across 3-7 clusters.

One of the most significant considerations in clustering is determining the optimal number of clusters. The Silhouette coefficient, which measures cluster cohesion and separation, provides valuable insights for this determination. The clustering solution with 4 clusters demonstrated a remarkable performance, exhibiting a Silhouette coefficient that was at least 28.3% higher than that of the solutions with 3 and 5-7 clusters (Figure 4A). This higher Silhouette coefficient indicates that the individual data within each cluster were, on average, more similar to each other and more dissimilar to individual data in other clusters in the 4-cluster solution compared to the other considered cluster formations. Furthermore, the DB index, which evaluates the compactness and separation between clusters, also provided additional support for the superiority of the 4-cluster solution. The DB index for the 4-cluster solution was at least 37.2% lower than that of the solutions with 3 and 5-7 clusters (Figure 4B). This lower index value signifies that the clusters in the 4-cluster solution were more well-defined and distinct from each other, reinforcing the efficacy of this particular clustering solution. Therefore, both the Silhouette coefficient and DB index confirmed that the 4-cluster configuration is the optimal choice for clustering.

**FIGURE 5:**
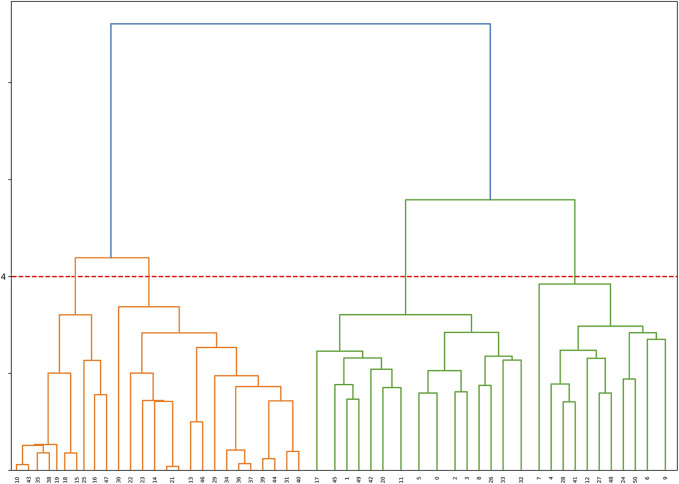
Dendrogram illustrating the hierarchical clustering of the dataset. The horizontal axis represents samples, and the vertical axis represents the dissimilarity between clusters. The red dashed line indicates the formation of 4 clusters based on the dendrogram’s structure, providing valuable insights into the underlying patterns within the data.

**FIGURE 6:**
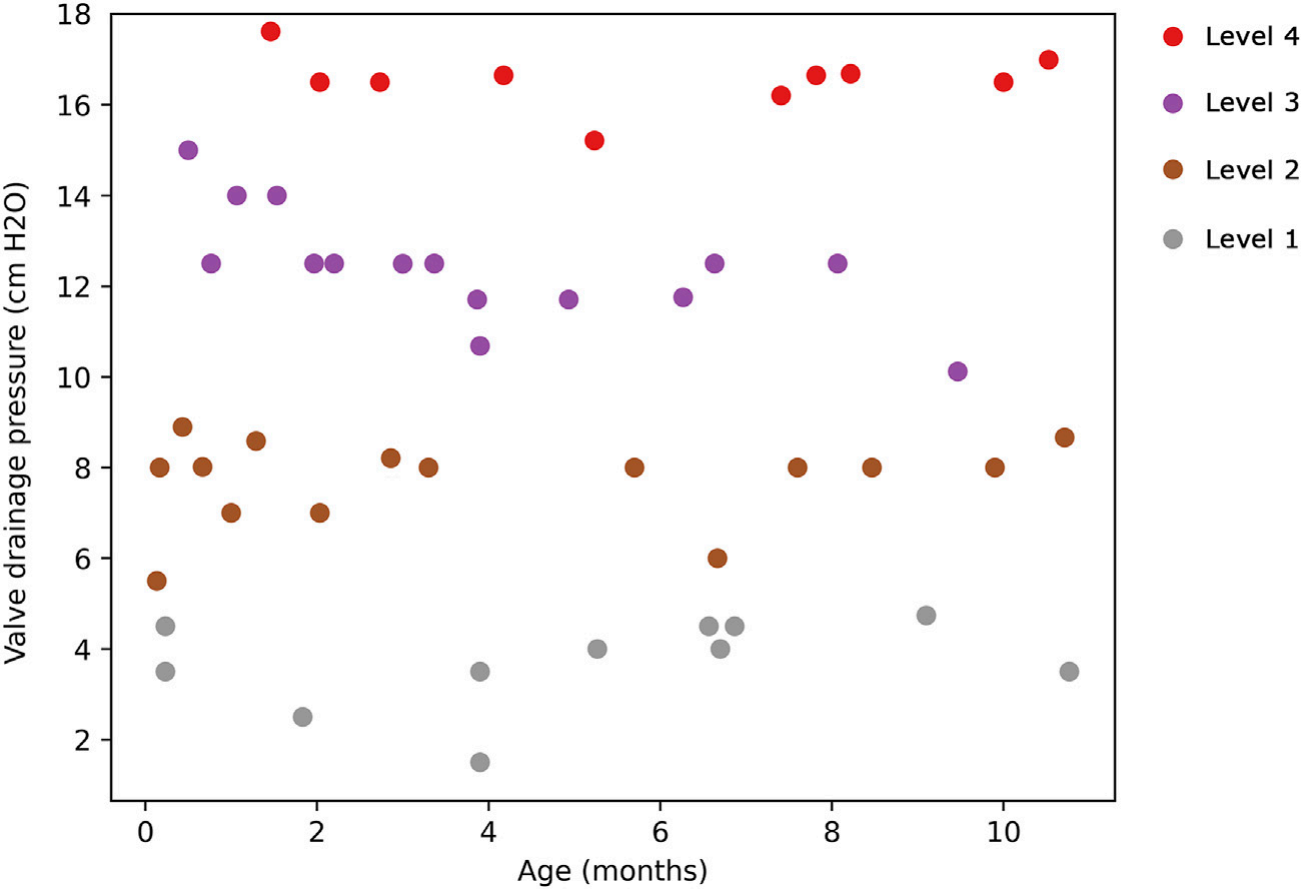
Scatter plot illustrating the hierarchical clustering of the dataset into 4 clusters. Cluster 1 (valve performance level 1) is denoted by gray points, Cluster 2 (valve performance level 2) by brown points, Cluster 3 (valve performance level 3) by purple points, and Cluster 4 (valve performance level 4) by red points.

**FIGURE 7:**
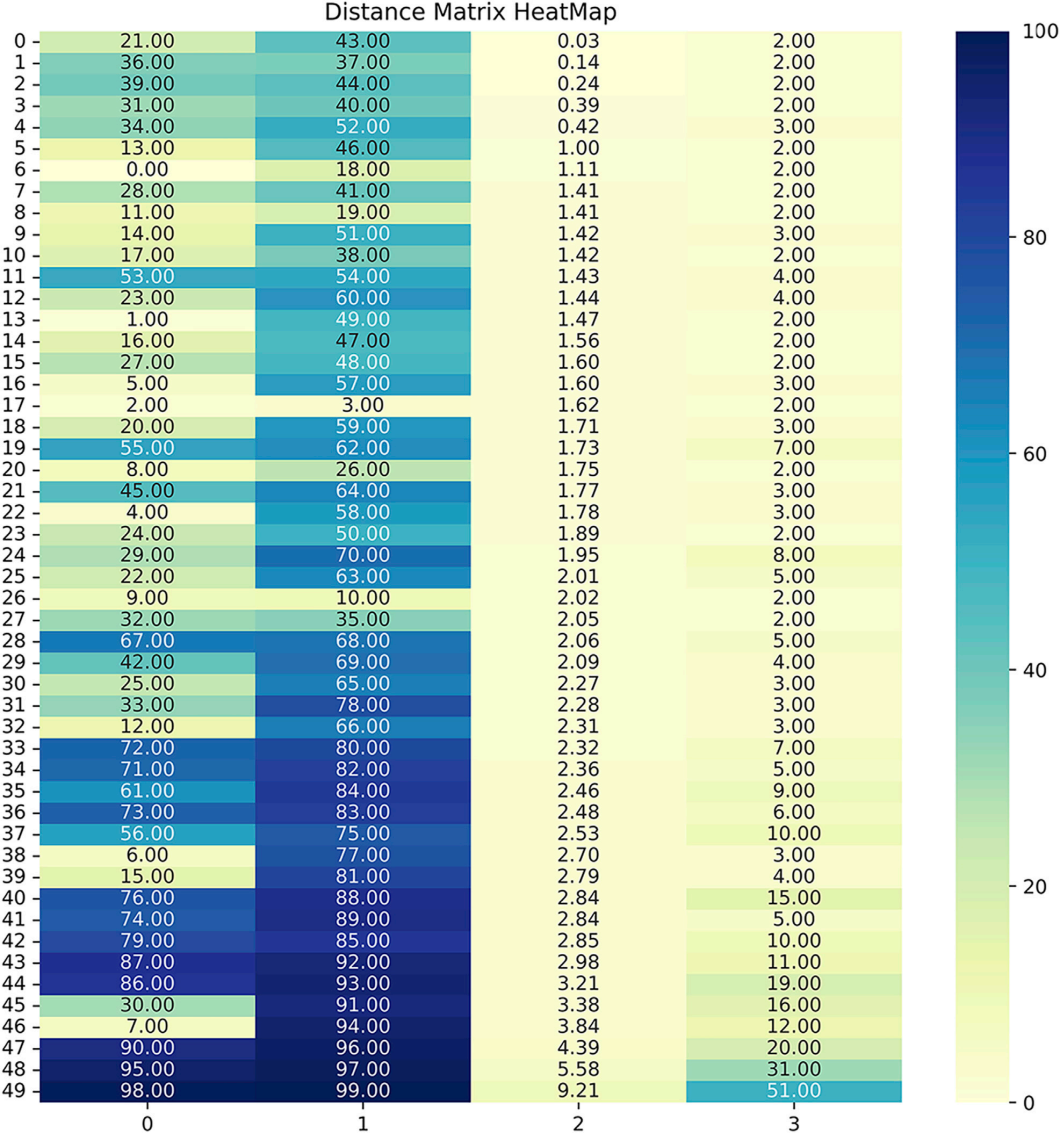
The distance matrix heatmap offers a visual summary of patient associations, guiding cluster analysis and highlighting distinct subgroups within the dataset. Darker colors, approaching 0, indicated smaller distances, showcasing highly similar or closely related patient pairs based on the measured features. Conversely, lighter colors, nearing 100, highlighted larger distances, emphasizing significant dissimilarities or distant relationships between patients. The 4 columns in the linkage matrix provided specific details: the first column contained the index of the first cluster merged in each iteration, the second column contained the index of the second cluster merged, the third column represented the distance between the merged clusters (calculated based on the chosen linkage criterion), and the fourth column denoted the number of original observations in the newly formed cluster.

**FIGURE 8:**
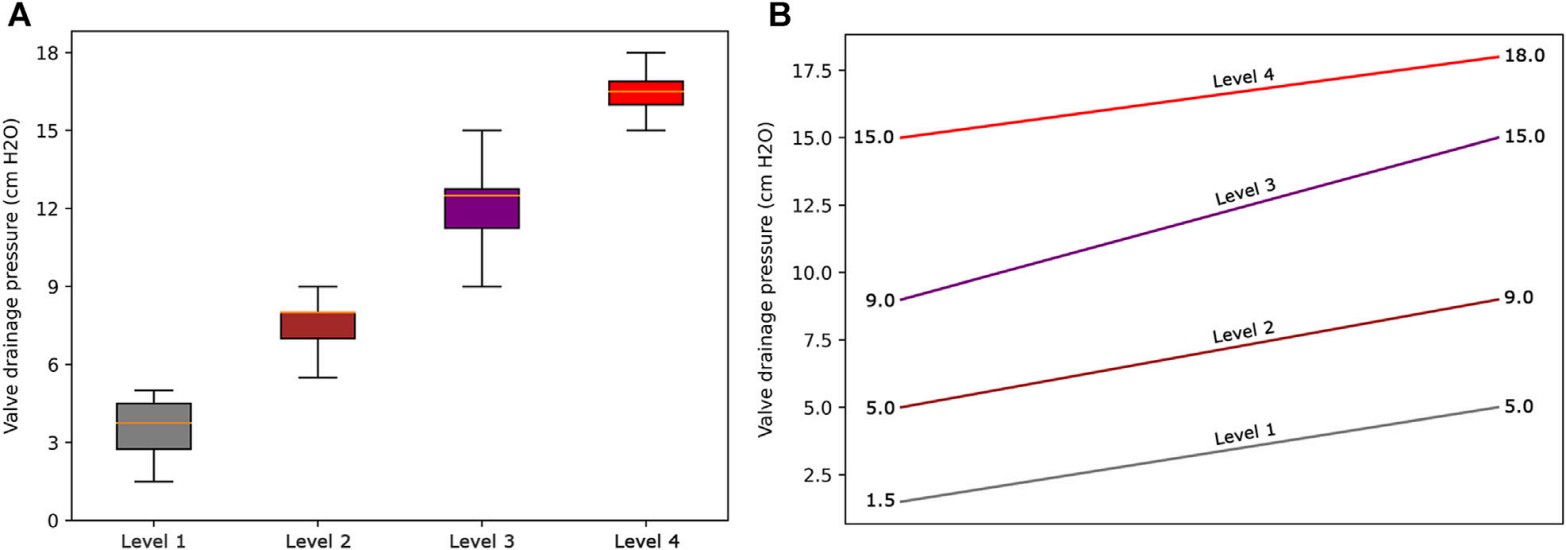
**(A)** Box plots to show the distribution of data for each valve performance level. **(B)** The range of valve drainage pressure is visualized for each performance level.

The resultant dendrogram, as depicted in Figure 5, provides a visual representation of the hierarchical clustering process, illustrating not only the arrangement of the data clusters but also the sequence and distance at which individual data points are merged into clusters. From this dendrogram analysis, we extracted four robust clusters (Figure 5: red dash line), labeled as Cluster 1 (valve performance level 1), Cluster 2 (valve performance level 2), Cluster 3 (valve performance level 3), and Cluster 4 (valve performance level 4). Each cluster encapsulates unique subsets of our data without any overlaps between the clusters. To enhance the interpretability of our clustering results, we generated a scatter plot (Figure 6). Each data point is represented, with distinct clusters color-coded. The plot effectively demonstrates the hierarchical clustering method’s ability to categorize the data into four clusters. Figure 7 showcases the distance matrix heatmap, which is central to elucidating the complex relationships among our patient cases based on pairwise dissimilarities. In this matrix, lighter shades indicate closer relationships, suggesting smaller distances between patient cases, whereas darker shades signify greater dissimilarities or larger distances. This matrix summarizes the hierarchical clustering iterations, detailing merged clusters, distances, and new cluster sizes. Based on the results from Figures 5–7, we organized and arranged the valve drainage pressure values for patients in each cluster in Figure 8. Box plots in Figure 8A highlight pressure differences across performance levels (levels 1–4). Each box represents the interquartile range, with the median shown inside, and whiskers display the pressure variability within clusters.

## Discussion

Previous research has highlighted the significant impact of optimizing valve performance levels, especially during the initial adjustment following the first shunt surgery, on shunt efficacy and the improvement of outcomes for infant hydrocephalus (Hall et al., 2021a; Bock et al., 2021). However, there are conflicts and contradictions about the optimal pressure range for each valve performance level, and consequently, the number of valve performance levels (Zemack et al., 2003; Yamada et al., 2022), among the large number of performance levels (ranging from 2 to 21) and a wide pressure range within each level (1–6 cm H_2_O) (Table 1). Therefore, finding the optimal thresholds for valve performance levels, which correlate more closely with the improvement of shunt outcomes, is of great importance. The present study aimed to optimize the number and range of valve performance levels to potentially assist neurosurgeons in enhancing shunt performance and improving clinical outcomes for infant hydrocephalus.

**FIGURE 9:**
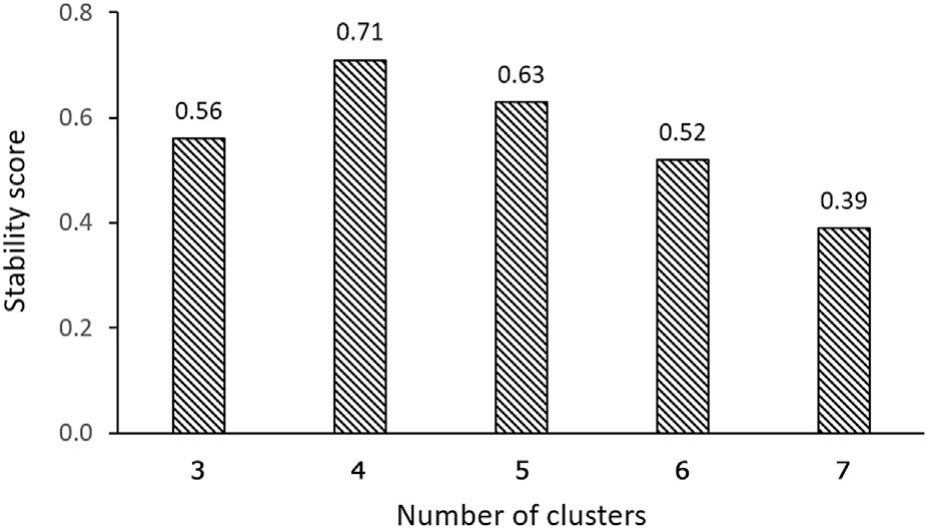
Cluster stability analysis based on the Jaccard index between 3-7 clusters.

Our findings demonstrate that, in terms of both intra-cluster similarity (indicated by the Silhouette coefficient) and inter-cluster dissimilarity (measured by the DB index), four valve performance levels outperform other alternatives (Figure 4). The results presented in Figure 4 also indicate that a three-level valve emerges as the next best alternative, as measured by intra-cluster similarity (Silhouette coefficient) and inter-cluster dissimilarity (DB index). However, practical and clinical considerations led us to focus solely on the first option (four-level valve). From a clinical and neurosurgical perspective, a three-level system is somewhat unconventional. To the best of our knowledge, none of the current shunts on the market utilizes a three-level system, which suggests a potential limitation in its functionality and its acceptance in current medical practice. Notably, the calculated valve drainage pressure ranges for each performance level were completely separated and distinct, and it was crucial for us to have clustering without any overlaps between different performance levels (Figures 6, 8). The repeatability of our results is essential for the implementation of our findings in a clinical study. Therefore, in addition to the acceptable results obtained from the Silhouette coefficient and the DB index for four clusters, cluster stability analysis becomes crucial for evaluating the repeatability, robustness, and reliability of our clustering results, particularly for relatively small data sizes. Employing bootstrapping techniques, we generated numerous iterations of resampled datasets, equal in size to the original dataset, to enable a rigorous and extensive assessment of the stability across our clustering analysis iterations. We employed the Jaccard index as our stability metric, enabling a precise comparison of clustering results from different resampled datasets. Our analysis revealed a notable Jaccard index of 0.71 for the 4-cluster, underscoring the reliability and repeatability of our chosen cluster and indicating that the identified clusters were not significantly influenced by random variations (Figure 9).

The cluster profile, as detailed in Table 3, encapsulates the diversity of our patient cohort across each cluster, distilling their multifaceted characteristics into four distinctive columns. Each row in this table represents a unique patient profile or feature. As per the results presented in Table 3, the mean (standard deviation) values of valve drainage pressure for performance levels 1, 2, 3, and 4 are 3.6 (0.86), 7.5 (0.62), 12.2 (0.99), and 16.7 (0.73) cm H_2_O, respectively. Understanding these distinct profiles is crucial as it enables clinicians and researchers to identify commonalities among patients within each cluster as patients within the same cluster might respond similarly to specific interventions. Figure 8B displays our proposed valve performance settings for infant hydrocephalus, featuring minimum and maximum values for the four performance levels (pressure range): 1.5–5 (3.5) cm H_2_O, 5–9 (4) cm H_2_O, 9–15 (6) cm H_2_O, and 15–18 (3) cm H_2_O, respectively.

**TABLE 3:** Cluster profile table. The values in the “Age” and “Valve drainage pressure” rows represent the mean values of age and valve drainage pressure in patients of those clusters. For other features, “0” indicates the absence of the feature for the majority of patients in the cluster, while “1” signifies the prevalence of the feature among patients in the cluster.

Valve performance levels (clusters)	Level 1	Level 2	Level 3	Level 4
Age (months)	5.1	3.4	3.4	5
Etiology: Brain/Skull Malformation	1	1	0	0
Etiology: Congenital/Idiopathic	0	0	0	1
Etiology: Cyst/Tumor	1	0	0	0
Etiology: Post-Infectious	0	0	0	1
Etiology: Post-hemorrhagic	1	1	1	1
Etiology: Spinal Disorder	0	1	0	0
Gender: Female	1	0	1	0
Gender: Male	0	1	0	1
Catheter Route: Frontal	0	0	1	0
Catheter Route: Occipital	0	1	0	1
Catheter Route: Parietal	1	0	0	0
Type: Communication	0	1	1	1
Type: non-communication	1	0	0	0
Anti-Siphon: No	0	1	1	1
Anti-Siphon: Yes	1	0	0	0
Programmable: No	1	1	0	0
Programmable: Yes	0	0	1	1
Valve drainage pressure (cm H_2_O)	3.6	7.5	12.2	16.7

Some widely-used valve brands, including Medtronic-Delta, also exhibit four valve performance levels, similar to our result. In the majority of common valve models such as Medtronic (Strata II, Strata MR II, Delta, and CSF Flow Control), Integra (Certas Plus), Codman (Uni-Shunt), Integra (Integra DP and Atlas), Natus (Novus), and Radionics (Equiflow), the pressure ranges in performance levels are not equal (Table 1), aligning with our finding. This finding may be attributed to the inherent variability in shunt outcomes among infant patients, where a consistent impact across equal pressure ranges was not always evident. On the other hand, certain shunt valve models, such as the Medtronic Strata II, Strata MR II, and Delta, as well as the Integra Certas Plus, define their valve drainage pressure ranges as less than or equal to 1 cm H_2_O per performance level (Table 1). In contrast, our findings—derived from patients without shunt failure and with improved shunt outcomes—revealed a broader pressure range of at least 3 cm H_2_O for each performance level. Although this differs from the narrower ranges set by these companies, it aligns more closely with the majority of other valve types (Table 1).

In our investigation of the etiology of infant hydrocephalus, we categorized etiological conditions in relation to different valve levels, as presented in rows 3-8 of Table 3. While these classifications yielded initial insights, they did not demonstrate a definitive separation of etiology across different valve levels, reflecting the inherent complexity of hydrocephalus etiology. This observation suggests that the relationship between specific valve settings and the underlying causes of hydrocephalus is not straightforward, indicating the need for more nuanced research approaches.

Our recent studies have emphasized the significance of time in the process of reducing ICP and enhancing intracranial compliance, and its impact on the improvement of clinical outcomes in gradual-onset brain disorders such as hydrocephalus (Gholampour, 2023a; Gholampour, 2023b). We have also highlighted the critical importance of precise control over the timing of CSF drainage following shunt surgery (Gholampour et al., 2022a; Gholampour et al., 2022c). We demonstrated that this control significantly influences the recovery behavior of brain tissue, thereby enhancing the efficiency of shunt performance and improving patient outcomes (Gholampour et al., 2022a). Our proposed optimization of the number and range of valve performance levels may also prove beneficial in enhancing control over the timing of CSF drainage. This, in turn, could facilitate the appropriate adaptation of brain tissue, ensuring proper brain recovery and increasing the likelihood of improved shunt outcomes.

This study strove to provide neurosurgeons with data-driven information about the optimized number and range of valve performance levels that may improve surgical outcomes in infant hydrocephalus. While our findings may offer valuable insights, it is important to acknowledge some limitations that should be addressed in future research. The specific geometry of shunt valves holds notable potential to influence their fluid resistance, subsequently impacting drainage flow rates within established pressure ranges. However, some literature suggests that this influence might not play a significant role in determining shunt outcomes (Hoshide et al., 2017). It is essential to recognize that while a linear relationship exists between the pressure and flow rate of CSF drainage, this relationship can vary between different valves, exhibiting diverse slopes. In our study, we have incorporated valve drainage pressure as one of the features in our machine learning analysis, thus indirectly accounting for its implication on flow rate. However, it is suggested for future studies to investigate the effects of various valve geometries on our optimization results. It is also important to highlight that this study specifically focused on pressure-controlled valves. However, we acknowledge the potential benefits of self-adjustable valves and recommend replicating our findings for self-adjustable valves in future research. Additionally, while our retrospective machine learning analysis is grounded in clinical data, it is important to note that this was a retrospective study. We recommend that future research adopt a prospective approach to apply our findings in a clinical trial with larger patient cohorts. This approach will facilitate the validation of the effectiveness of the optimization results and enable the assessment of the correlation between our proposed valve levels and the varying levels of clinical outcomes in both infant and older pediatric patients with hydrocephalus across diverse clinical scenarios. Moreover, this was an unsupervised (non-classification) machine learning analysis; therefore, focusing on patients with unimproved outcomes in future studies could be beneficial for conducting error analysis.

## Conclusion

In this study, we applied unsupervised hierarchical machine learning analysis to cluster infant hydrocephalus patients based on features such as age, gender, hydrocephalus etiology (six categories), type of hydrocephalus (two categories), shunt catheter route (three categories), anti-siphon condition, programmable status, number of valve adjustments, and valve drainage pressure. The evaluation of the Silhouette coefficient, DB index, and cluster stability analysis using the Jaccard index unequivocally established that the optimal number of valve performance levels for infant hydrocephalus was four: 1.5–5.0, 5.0–9.0, 9.0–15.0, and 15.0–18.0 cm H_2_O. The recommendation of our optimized number and range of valve performance levels holds the potential for neurosurgeons to improve shunt outcomes in infant hydrocephalus, ultimately enhancing the quality of life for these patients.

## Data Availability

The original contributions presented in the study are included in the article, further inquiries can be directed to the corresponding author.

## Abbreviations

CNS, Central nervous system; CSF, Cerebrospinal fluid; ICP, Intracranial pressure; IRB, Institutional review board; DB, Davies-Bouldin; PCA, Principal component analysis.
